# Efficacy of novel agents in patients with nephropathy associated with POEMS syndrome

**DOI:** 10.1007/s11255-022-03120-9

**Published:** 2022-02-08

**Authors:** Shuiqin Cheng, Li Huang, Wenjing Fan, Dandan Liang, Xiaodong Zhu, Song Jiang, Yongchun Ge

**Affiliations:** National Clinical Research Center of Kidney Diseases, Jinling Hospital, Nanjing University School of Medicine, #305 East Zhongshan Road, Nanjing, 210016 China

**Keywords:** Nephropathy associated with POEMS syndrome, Novel agents, Bortezomib and dexamethasone, Repeat renal biopsy, Efficacy

## Abstract

**Objective:**

To evaluate the clinical characteristics and outcomes of patients with nephropathy associated with POEMS syndrome who received novel agents in combination with dexamethasone therapy, and renal pathological changes based on repeat biopsy in some patients after these novel-agent-based therapies.

**Methods:**

The records of patients with nephropathy associated with POEMS syndrome in a single hospital from May 2017 to February 2021 were retrieved and studied in detail. All the patients received four cycles of initial novel-agent-based regimens such as bortezomib and dexamethasone (BD) or thalidomide plus dexamethasone (TD) or lenalidomide plus dexamethasone (RD) treatment. We further evaluated the pathological efficacy of these novel agents by repeat renal biopsy.

**Results:**

Twelve patients with an average age of 48.6 ± 8.3 years diagnosed with nephropathy associated with POEMS syndrome were enrolled in this study. The duration from disease onset to renal biopsy was 28(8.3 ~ 54.5) months. All patients achieved good clinical responses in different degree after four cycles of initial novel agents in combination with dexamethasone therapy. After the treatment with novel-agent-based regimens, the levels of proteinuria decreased in most patients and were negative in five patients. The levels of serum creatinine (SCr) decreased in ten patients. Serum M protein was negative in four patients and still positive in the other eight patients. The levels of serum vascular endothelial growth factor (VEGF) were detected in seven patients, which were all decreased. The levels of interleukin-6 (IL-6) were detected in eight patients, which were also decreased. Repeat biopsies were performed after four cycles of novel-agent-based therapies in four patients who were all treated with BD treatment. Mesangiolysis, mesangial cells proliferation, endothelial cells proliferation, subendothelial space widening and acute renal tubulointerstitial lesions improved, the chronic renal tubulointerstitial lesions were stable.

**Conclusions:**

Novel agents improved clinical manifestations in patients with nephropathy associated with POEMS syndrome. In addition, novel-agent-based regimens such as BD treatment improved renal pathological manifestations, which suggested that novel agents could improve renal prognosis of the patients from the perspective of renal pathology.

## Introduction

Polyneuropathy, organomegaly, endocrinopathy, M-protein, and skin changes (POEMS) syndrome, is a rare multisystemic disease secondary to a plasma cell dyscrasia [1], the diagnosis of POEMS syndrome do not require the presentation of all these symptoms, but many other clinical criteria are included in the diagnosis of POEMS, such as castleman disease, sclerotic bone lesions, vascular endothelial growth factor(VEGF) elevation, extravascular volume overload, papilledema, thrombocytosis/polycythemia, etc.[2]. Renal involvement is not rare in Chinese patients with POEMS syndrome which had a proportion of 37% [3]. Nephropathy associated with POEMS syndrome was characterized as POEMS syndrome diagnosed and renal biopsy manifested as membrano-proliferative glomerulonephritis (MPGN)-like lesions, microangiopathic lesions, glomerular endothelial cell proliferation, and mesangiolysis [4]. Ye et al. [4] retrospectively reviewed 299 Chinese patients diagnosed with POEMS syndrome from 2000 until 2014, which indicated that renal impairment was a common complication of POEMS syndrome and was also an independent risk factor of survival, but could be reversed with effective therapy in most cases.

In POEMS syndrome, the primary aim of treatment is to target plasma cells and suppress the production of the toxic monoclonal protein. Autologous stem cell transplantation (ASCT) [5], melphalan [6], immunomodulatory agents(thalidomide/lenalidomide) [7, 8] and bortezomib, [9] etc., had good therapeutic effects in clinical application. Treating POEMS syndrome with these therapies is particularly tempting, not only because of their excellent action against plasma cells, but also owing to reversion of renal impairment. However, there were no series of studies observed whether the renal pathological lesions could improve after treatment in patients with nephropathy associated with POEMS syndrome. Herein, we retrospectively evaluated clinicopathological characteristics and outcomes of novel-agent-based regimens in patients with nephropathy associated with POEMS syndrome who had repeat renal biopsy.

## Patients and methods

### Patients

Patients diagnosed with POEMS syndrome according to published diagnostic criteria [10] and biopsy-proven nephropathy were enrolled in the National Clinical Research Center of Kidney Diseases, Jinling Hospital from May 2017 to February 2021. The criteria for inclusion in the study were as follows: (1) age > 18 years; (2) POEMS syndrome diagnosed definitely; (3) biopsy-proven nephropathy associated with POEMS syndrome; (4) received four cycles of novel-agent-based regimens such as bortezomib and dexamethasone (BD) or thalidomide plus dexamethasone (TD) or lenalidomide plus dexamethasone (RD) treatment hospitalized in our center. We performed repeat biopsy for four patients after the end of last cycle of treatment within 1 week, to evaluate the renal pathological changes. The criteria for exclusion were as follows: (1) Patients who used novel agents within 24 weeks and dexamethasone within 4 weeks of receiving renal biopsy; (2) females who were pregnant or have pregnancy desire; (3) malignancy or cardiac failure, liver failure, bleeding ulcers and so on; (4) patients who had mental disorder.

This study was approved through the local ethics committee of Jinling Hospital (IRB: 2017NZYW-105-018). All the enrolled patients signed the consent of renal biopsy before renal biopsy was performed.

### Measurements and variable definitions

The gender, age, the duration of renal diseases, and the clinical manifestation were recorded. The levels of 24-h proteinuria, urinary sediment red blood cell (RBC), serum biochemical parameters, M-protein, VEGF, interleukin-6 (IL-6) were all recorded at baseline and after novel-agent-based regimens. Serum VEGF level was tested using a human VEGF commercial ELISA kit at a local research facility, manufacturer’s reference range of serum VEGF level was 0–142 pg/ml.

Proteinuria was defined as urinary protein > 0.4 g/24 h. Hematuria under light microscopy was defined as RBC count > 12/ul in the urinary sediment. Complete renal remission was defined as proteinuria < 0.4 g/24 h, serum albumin ≥ 35 g/L and serum creatinine (SCr) ≤ 110 μmol/L. Partial renal remission was defined as proteinuria > 0.4 g/24 h and < 3.5 g/24 h with a ≥ 50% decrease of proteinuria from baseline, SCr was stable or of increase < 30% from baseline. No renal response was defined as proteinuria > 3.5 g/24 h and/or SCr increased ≥ 30% from baseline [11].

### Renal pathology

Percutaneous renal biopsies were performed under the guidance of ultrasound at baseline and the end of the fourth cycle of novel-agent-based regimens within 1 week. All of the cases were processed using light, immunofluorescence, and electron microscopy. The renal biopsy procedure was specified as follows: the samples were embedded in paraffin and were sectioned at 1.5 μm, followed by hematoxylin–eosin, Masson, periodic acid-Schiff, and periodic acid-silver methenamine staining. There were more than ten glomeruli observed under the light scope in each biopsy specimen. The tissue samples were sectioned in frozen conditions, cut at 4 μm for immunofluorescence staining of IgG, IgA, IgM, C3, C1q, κ and, λ-light chain using polyclonal FITC-conjugated antibodies. Deposits and distribution were observed. Electron microscopy observations were performed with the Hitachi 7500 electron microscope.

MPGN-like lesions are proliferation of mesangial and endothelial cells with basement membrane thickening and stratification was similar to MPGN by light and electron microscopy. Mesangiolysis is a glomerular process leading to lesions of continuous endothelial cells and glomerular mesangial cells, endothelial cell proliferation, endothelial swelling and subendothelial widening, endothelial injury caused mesangial interposition, finally mesangial proliferation and mesangial sclerosis [12]. Glomerular endothelial cell proliferation was defined as the endothelial cell number was increased along with enlargement of the subendothelial spaces [13], glomerular subendothelial widening was defined as formation of new layer(s) of basal lamina like double contour basement membrane in capillary of the glomerulus[14]. Three grade evaluation in semi-quantitative analysis based on the percentage of the glomeruli compartment and recorded was mild (1 + , < 25%), moderate (2 + , 26–50%), and severe (3 + , > 50%) [15, 16]. The renal tubulointerstitial lesions were divided into chronic and acute lesions. Chronic renal tubulointerstitial was defined as interstitial fibrosis and tubular atrophy (IFTA). Acute renal tubulointerstitial lesion was defined as acute tubular cell necrosis (ATN) and interstitial edema. We determined the area of IFTA and ATN on digitized images using the Leica Aperio AT Turbo Imagescope software (version 12.3). The percentages of IFTA area and ATN area relative to the cortical area of the image field were calculated [17]. Interstitial inflammatory cell infiltration was evaluated semi-quantitatively based on the percentage of the tubulointerstitial compartment and recorded as mild (1 + , < 25%), moderate (2 + , 26–50%), and severe (3 + , > 50%). All kidney biopsy specimens were reviewed by two renal pathologists for quantifying the specific lesions.

### Treatment strategy

Twelve patients of nephropathy associated with POEMS syndrome were initial treated with BD (five patient), RD (two patients) and TD (five patients) for four cycles. The BD treatment as follows: bortezomib 1.3 mg/m^2^ hypodermic injection and dexamethasone 40 mg intravenously on days 1, 4, 8, and 11 of the 21-day cycle. The RD treatment as follows: Lenalidomide 10–25 mg/day for 21 days followed by 7 days rest plus dexamethasone 40 mg/week through oral administration. The TD treatment as follows: Thalidomide 100–200 mg/day for 21 days followed by 7 days rest plus dexamethasone 40 mg/week through oral administration.

### Statistical analysis

Statistical analysis was performed using the statistical software SPSS 26.0 (SPSS Inc, Chicago, IL, USA). Parametric data consistent with normal distribution were expressed as means ± standard deviation. Nonparametric data were expressed as median (inter quartile range).

## Results

### Baseline characteristics and clinical responses for 12 patients

We enrolled 12 patients (6 males and 6 females) diagnosed with nephropathy associated with POEMS syndrome. The average age was 48.6 ± 8.3 years and duration from disease onset to biopsy was 28(8.3 ~ 54.5) months. All the 12 patients had nonspecific symptoms such as emaciation and fatigue. They had peripheral neuropathy manifestation of feet numbness or tingling, and their physical examination showed decreased muscle strength and weakened tendon reflex. Electromyography examination indicated neurogenic damage in nine patients, neurogenic with myogenic damage in three patients. Seven patients manifested hypertension. Serum M protein on serum protein electrophoresis was detected in all the 12 patients, the type of M protein was IgA-λ in 7 patients, IgG-λ in 3 patients and IgG-κ in the other 2 patients. All the 12 patients had organomegaly, in which 8 patients had renal enlargement, 6 patients had hepatomegaly, 7 patients had splenomegaly, and 4 patients had lymphadenopathy. Endocrinopathies were found in 10 patients, including hypothyroidism in 8 patients, diabetes mellitus in 4 patients. Hyperpigmentation was found in all the 12 patients, in which 8 patients had white nails and 2 patients had skin cherry angiomata. All the 12 patients were screened for bone by X-ray, and sclerotic bone lesions were observed in 3 patients. Bone marrow cytology test was normal in all the 12 patients. The levels of serum VEGF concentration were detected in 9 patients elevated (602.9 ~ 4269.3 pg/ml).

Twelve patients achieved good clinical responses for four cycles of initial novel agents in combination with dexamethasone therapy. After treatment, feet numbness or tingling improved in different degree in the all patients. Complete renal remission in two patients, partial renal remission in eight patients, no renal response in the other two patients. The levels of proteinuria decreased in different degree in most patients and were negative in 5 patients after treatment, the levels of urinary sediment RBC decreased in 11 patients, the levels of SCr decreased in 10 patients. Patient 3 who received hemodialysis due to azotemia and volume load at baseline was free from dialysis after four cycles of the BD treatment. Serum albumin was raised in the 11 patients. Serum M protein was negative in four patients and still positive in the other eight patients. The levels of serum vascular endothelial growth factor (VEGF) were detected in seven patients, which all decreased. The levels of IL-6 were detected in eight patients, which were also decreased. The clinical characteristics of these patients were summarized in Table 1.

**Table 1:** Baseline characteristics and clinical responses for four cycles of initial novel-agent-based therapies in patients with nephropathy associated with POEMS syndrome

Patient	Age/gender	Disease onset to biopsy (months)	Treatment	Urinary protein(g/24 h)		Urinary sediment RBC(/μl)		Serum albumin(g/L)		SCr(μmol/L)		M protein		VEGF(pg/ml)		IL-6(ng/L)	
				Baseline	After treatment	Baseline	After treatment	Baseline	After treatment	Baseline	After treatment	Baseline	After treatment	Baseline	After treatment	Baseline	After treatment
1	55/Male	71	BD	1.89	0.77	57.2	11.9	44.2	44.8	65.4	61.0	IgA-λ	IgA-λ	3581.4	303.8	ND	ND
2	45/Male	31	BD	1.08	1.05	81.6	5.1	39.4	45.2	118.5	87.5	IgG-λ	Neg	2876.3	45.21	15.4	5.09
3	52/Female	2	BD	0.59	0.33	71.7	4.3	33.5	33.8	348.3	205.1	IgA-λ	IgA-λ	4269.3	455.1	44	5.79
4	54/Female	37	BD	0.86	0.34	21.9	4.2	38.9	42.3	91.9	86.6	IgA-λ	Neg	2026.7	179.3	4.74	1.5
5	53/Male	25	BD	0.72	0.22	165.2	12	35	36.9	217	244.9	IgA-λ	IgA-λ	1436.8	386.6	44.62	28.78
6	49/Female	8	RD	0.35	0.23	7.6	8.5	34.5	45.4	130.8	115.8	IgG-λ	IgG-λ	2675.4	ND	26.24	15.14
7	36/Female	47	RD	0.95	0.56	40.5	18.3	40	49.1	175	139.7	IgA-λ	Neg	ND	ND	39.76	8.49
8	46/Male	57	TD	1.88	1.42	77.8	40.3	28.1	41.5	117.6	95.5	IgG-κ	IgG-κ	1563.1	120.3	ND	ND
9	51/Female	19	TD	1.61	0.79	34.2	13.4	36.1	43.2	83.1	72.3	IgA-λ	IgA-λ	ND	ND	ND	ND
10	53/Male	65	TD	0.36	0.24	8.3	0.5	42.7	44.5	106.1	91.9	IgG-κ	IgG-κ	602.9	42.1	ND	ND
11	59/Female	9	TD	7.46	2.33	80	11	22	32.3	90	71	IgG-λ	IgG-λ	ND	ND	7.46	4.63
12	30/Male	5	TD	1.48	1.35	11	29.7	34.2	32.1	150	216.3	IgA-λ	Neg	1115.5	ND	26.86	23.02

*SCr* serum creatinine, *F* female, *M* male, *BD* bortezomib and dexamethasone, *RD* lenalidomide plus dexamethasone, *TD* thalidomide plus dexamethasone, *VEGF* vascular endothelial growth factor, *IL-6* interleukin-6, *ND* no data, *Neg* negative

### Renal pathological manifestations before and after novel-agent-based therapies

We performed renal biopsy in 12 patients who received 4 cycles of novel-agent-based therapies. Before treatment, immunofluorescence staining of IgG, IgA, IgM, C3, C1q, κ, and λ-light chains were all negative. Mesangiolysis and proliferation of mesangial and endothelial cells were observed in the all four patients. Chronic renal tubulointerstitial lesion calculated as IFTA percentage was ranged from 18.7 to 38.4%, and acute renal tubulointerstitial lesion calculated as ATN percentage was ranged from 4.8 to 58.6%. All the four patients had arteriole dissepiment thickening, arteriole stenosis, and/or arteriole hyalinosis, although only two patients manifested hypertension. Subendothelial space widening without dense deposition was also pointed out under electron microscopy.

**Fig. 1:**
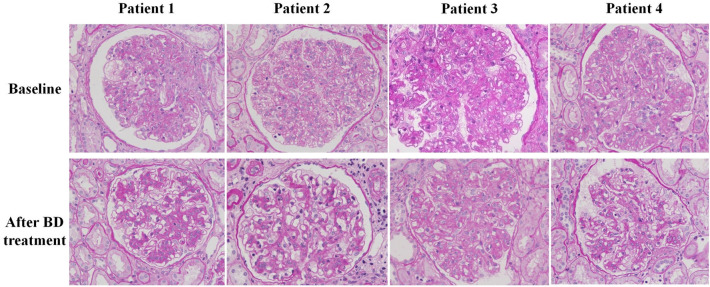
Pathological features of baseline and repeat biopsies. Mesangiolysis, as well as mesangial and endothelial cells proliferation improved and replaced by increased mesangial matrix after BD treatment compared with baseline (periodic acid Schiff stain, × 400)

**Fig. 2:**
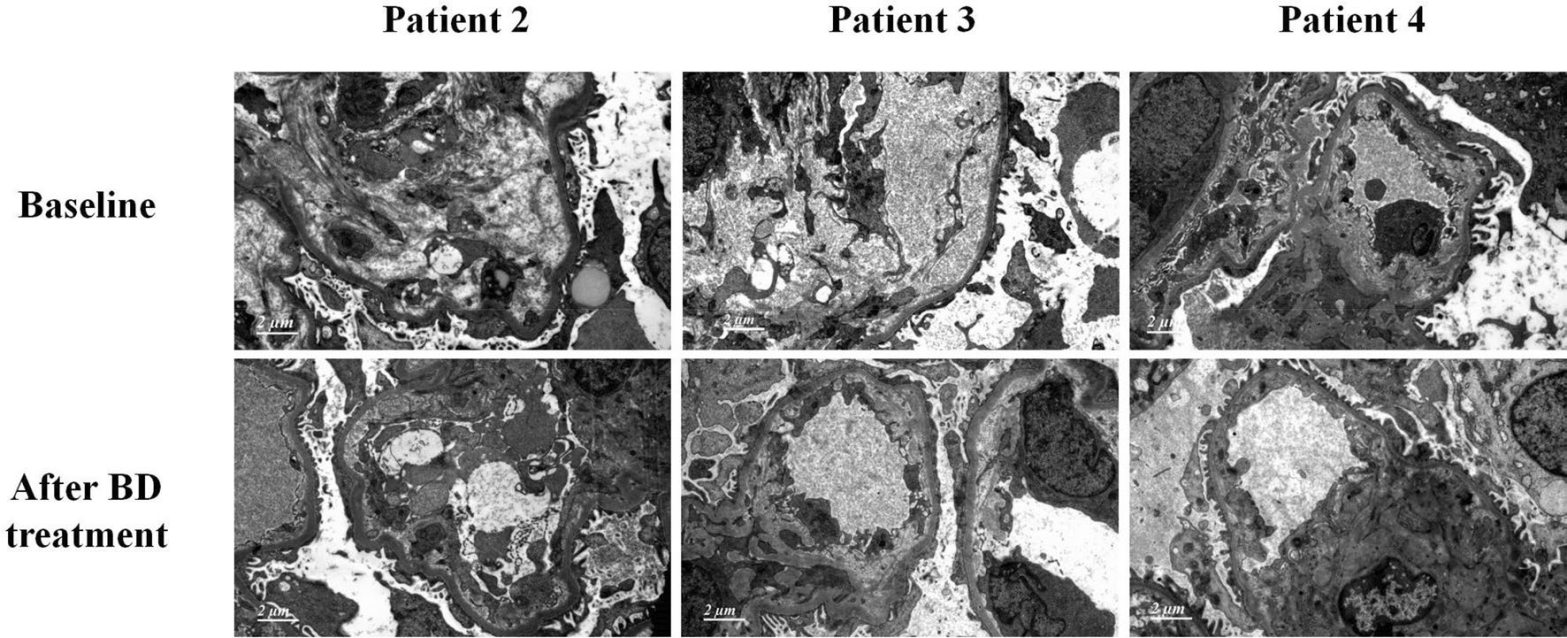
Glomerular ultrastructure of baseline and repeat biopsies. Subendothelial space widening significantly improved after BD treatment compared with baseline (magnification, × 10,000)

Repeat renal biopsies were performed after four cycles of novel-agent-based therapies in the four patients. Pathological features of repeat biopsies indicated that the mesangiolysis, as well as mesangial and endothelial cell proliferation all improved after four cycles of treatment. The area of acute renal tubulointerstitial lesion decreased to 0–36.9%. The chronic renal tubulointerstitial lesion was stable, IFTA percentage was ranged between 19.2 and 39.2%, because the chronic tubulointerstitial fibrosis cannot be reversed. Renal arteriole lesions were stable and did not improve or aggravate, because the arteriole lesions were predominately chronic. The ultrastructure of glomerulus was observed under electron microscopy. No glomerular was found in patient one in the samples of both baselines and repeat biopsy under electron microscopy. The observation of glomerular ultrastructure in the other three patients indicated that mesangiolysis disappeared, replaced by increased mesangial matrix. Subendothelial space widening significantly improved. The erythrocyte and endothelial cells were observed in subendothelial space in patient three at baseline biopsy and disappeared at repeat biopsy, which suggesting that the glomerular endothelial cells were severely damaged at baseline and these lesions can be restored after BD treatment. The proportion of podocyte foot process fusion in glomerulus was 10–15%, which was not significantly changed after treatment. Renal pathological characteristics of these four patients after four cycles of novel-agent-based therapies were summarized in Table 2, and the pathological features before and after novel-agent-based therapies were shown in Figs. 1, 2.

**Table 2:** Pathological manifestations of four patients who had repeat renal biopsy before and after 4 cycles of BD treatment

	Patient 1		Patient 2		Patient 3		Patient 4	
	Baseline	After treatment	Baseline	After treatment	Baseline	After treatment	Baseline	After treatment
Light microscopy								
Numbers of glomeruli	19	56	31	30	27	29	37	40
Numbers of globally sclerotic glomeruli	1	13	4	1	1	0	2	2
Mesangiolysis	Diffuse	Focal	Diffuse	Only 1 lesion	Diffuse	Focal	Diffuse	Focal
Endothelial cells proliferation	3 +	1 +	3 +	1 +	3 +	2 +	3 +	1 +
Mesangial cells proliferation	3 +	2 +	3 +	1 +	3 +	2 +	3 +	1 +
Mesangial matrix increase	1 +	2 +	1 +	2 +	2 +	2 +	1 +	2 +
Acute tubulointerstitial lesion	4.8%	0%	39.7%	5.2%	58.6%	36.9%	32.1%	9.7%
IFTA	21.3%	29.7%	38.4%	39.2%	18.7%	19.2%	29.8%	28.9%
Interstitial inflammatory cell infiltration	1 +	1 +	2 +	1 +	3 +	2 +	2 +	1 +
Electron microscope								
Subendothelial space widening	ND	ND	3 +	1 +	3 +	1 +	2 +	1 +

*ND* no data, *IFTA* interstitial fibrosis and tubular atrophy

### Side effects of novel agents

Twelve patients finished four cycles of novel-agent-based therapies without severe adverse events. Mild thrombocytopenia was observed in one patient. Gastrointestinal symptoms including nausea, poor appetite or constipation were observed in two patients. Elevation of SCr concentration was presented in three patients. Although the most common non-hematologic toxicity of bortezomib was peripheral neuropathy, feet numbness or tingling were significantly improved in five patients. Feet tingling was aggravated in one patient during the BD treatment. All the complications mentioned above can be well-tolerated, and improved after the completion of the fourth cycle.

## Discussion

POEMS syndrome is a multisystemic disease secondary to plasma cell dyscrasia. Diagnostic criteria for POEMS were first proposed in 2003 [18] and were revised after the diagnostic value of serum VEGF concentration was confirmed in 2007 [19]. According to the latest diagnostic criteria of POEMS syndrome[2], the clinical features have been classified into mandatory criteria, major criteria, and minor criteria, of all the patients had two mandatory major criteria, at least one other major criterion and at least one minor criterion, so the patients in our center diagnosed with POEMS syndrome. The clinical course of POEMS syndrome is usually chronic. Keddie S et al. [20]demonstrated that one hundred patients of POEMS syndrome had a 5-year and 10-year survival of 90% and 82%. Some studies [7, 21–24] indicated that the treatment targeting plasma cells including melphalan, thalidomide, bortezomib and ASCT, lenalidomide have significantly improved the clinical manifestations and significantly decreased the levels of serum VEGF.

VEGF could be produced by plasma cells and proliferating endothelial cells [25]. It can explain elevated VEGF in TMA pathogenesis, because the prominent pathological feature of TMA is small vascular endothelial cell lesion, which is characterized by endothelial cell proliferation and swelling. Our study indicated that the M protein became negative in four patients after novel agents in combination with dexamethasone treatment, serum VEGF were measured decreased significantly in two of the four patients, and the mesangiolysis and endothelial cell proliferation reversed better in the four patients who had BD treatment by repeat biopsies. These results suggested BD treatment could significantly inhibit plasma cells and endothelial cells proliferation of nephropathy associated with POEMS syndrome, ultimately, it leaded to a decrease of VEGF of these patients. Besides, proliferating glomerular endothelial cells and subendothelial space widening with deposition of plasma composition were observed in these four patients at baseline. In patient 3, the proliferation of glomerular endothelial cells was the most severe, the VEGF concentration was the highest, and erythrocyte and endothelial cells were even observed in subendothelial space, these phenomena indicated that elevated VEGF may be associated with the proliferation of glomerular endothelial cells. After novel-agent-based therapies, subendothelial space widening significantly improved, the levels of serum VEGF decreased. Exposure of human vascular endothelial cells to VEGF increased the expression of IL-6 in plasma cell disorders [25]. The high titer of serum VEGF and IL-6 could explain common characteristic endovascular lesions of POEMS syndrome [26]. In eight patients whose IL-6 were measured, the levels of IL-6 decreased after treatment especially BD treatment, suggesting that the levels of IL-6 also associated with the clinical and renal response to these treatments.

Although renal involvement was not included in the diagnostic criteria of POEMS syndrome, it was not unusual and its manifestations were protean. A previous study has demonstrated that proteinuria and microhematuria was present in 20% and 15% of the Chinese patients with POEMS syndrome. Nine percent of these patients had both proteinuria and microhematuria simultaneously, and 37% of these patients had renal impairment [3]. Only a few studies evaluated the renal pathological characteristics of patients with nephropathy secondary to POEMS syndrome were reported [3, 4, 27], mesangial and endothelial cell proliferation, endothelial cells swollen, capillary lumens occluded, thickening of the glomerular basement membrane, and double contour formation were the most common glomerular changes reported in these studies. Besides, the IFTA and arterioles hyalinosis, arteriolar wall thickening and luminal occlusion were occasionally observed. Immunofluorescent staining showed no deposition of immunoglobulin or complement in general. However, light chain deposit disease, immunotactoid glomerulopathy and IgG4 producing POEMS syndrome were also described in case reports [28–30], we speculated that these lesions may be induced by monoclonal immunoglobulins produced by plasma cells in patients with POEMS syndrome. Even though the clinical and pathological characteristics of renal involvement in patients with POEMS syndrome have been reported in a few previous studies, it is far from clear that whether the lesions of glomerular, tubulointerstitial and arterioles can be reversed or progressed after the effective treatment. Although only four patients had repeat renal biopsy in the present study, with preciousness that we are the first to evaluate the therapeutic effects of novel agents in patients with nephropathy associated with POEMS syndrome by repeat renal biopsy, aiming at clarifying the renal pathological feature changes after treatment. The results of the present study indicated that the mesangiolysis, proliferation of mesangial and endothelial cells, and acute renal tubulointerstitial lesions of the four patients significantly improved and partially reversed, but these lesions cannot be reversed to normal, and mesangial matrix increased, even if the proteinuria and hematuria had different degrees of remission, which suggested that the four cycles of novel-agent-based therapies were not enough during the disease course. Thereafter, TD or RD were prescribed to the four patients as maintenance treatment after four cycles of initial therapies.

In this study, bortezomib, thalidomide and lenalidomide were treated in patients with nephropathy associated with POEMS syndrome. They have the advantages of being effective, easily available clinically, and with the implementation of medical insurance policy, the cost gradually decreased. Bortezomib is administered intravenously, while thalidomide and lenalidomide are more convenient to take orally. Bortezomib has the advantages of not requiring dose adjustment according to renal function and not having the risk of thrombosis, while thalidomide and lenalidomide need dosage adjustment for renal impairment and have risk of thrombosis [9]. Lenalidomide had broader immunomodulatory and stronger anti-tumor effects than thalidomide, and had lower neurotoxic and cardiotoxic reactions than thalidomide, lenalidomide could be used in POEMS syndrome patients refractory to thalidomide [31]. BD treatment is the first-line therapy for patients with POEMS syndrome especially who had renal impairment, and it showed a high clinical remission rate as an induction therapy [9].

This study was limited by the following factors: (1) Only 12 patients were enrolled, and only four patients received repeat renal biopsy after 4 cycles of novel-agent-based therapies; (2) we did not observe the glomerular pathological feature under electron microscope in patient one because of the reasons for pathological sampling; (3) the follow-up time of novel-agent-based therapies was 12 weeks, the efficacy needed to be further evaluated.

## Conclusion

In the present study, our results indicated that novel agents could improve clinical outcomes in patients with nephropathy associated with POEMS syndrome. In addition, the therapy of BD treatment could improve renal pathological manifestations, which suggested that novel agents were efficacy and could improve renal prognosis of the patients.

## Declarations

### Human and animal participants

All the human samples used in the study were approved by the Local Ethics Committee of Jinling Hospital.
